# On Quackery, and the Most Effectual Means of Checking Its Dangerous Progress

**Published:** 1799-06

**Authors:** 


*To the Editors of the Medical and Physical Journal,*

GENTLEMEN,

I HOPE the inclosed observations, on the subject of quackery, will not be considered as improper for insertion in the next Number of your valuable and truly interesting Medical Journal.

I am, Gentlemen,

With respect, yours, &c.

*April* 24th, 1799.  ALIQUIS.

### *On Quackery, and the most effectual Means of checking its dangerous Progress.*

IT is not an easy matter to determine, what mode of administering medicine comes within the description of quackery; it can hardly be extended to all advertised remedies, because in that case, we must involve many prescriptions of eminent and regular physicians, who, actuated by various motives, have communicated their discoveries to mankind in this way; or the fruits of whose labours in the invention and application of new medicines, have found their way into the world by accidental means, without their concurrence.

There are also some medicines which have been reported to be so highly efficacious in the cure of particular disorders, that the legislature have thought fit to pay the inventors a reward for their ingenuity, and to publish the compositions of their nostrums for the benefit of mankind.

But we do not apprehend that either of those classes come properly under the denomination of quackery. Where the component parts of a medicine are publicly known, as well as the exact manner of preparing it, or where it comes forth sanctioned by the name of a regular physician, of known and approved abilities and integrity, however it is promulgated, or in whatever way the diseased are supplied with it, we do not apprehend, in either case, the medicine ought to be considered as quackery, or that its value is at all lessened by being vended at such a price as renders it attainable by the afflicted in every station of life, or degree of circumstances *.

But

* Although we can by no means subscribe to the limited definition of quackery given by our correspondent, nor can it be disputed, that Nostrums in general, whether encouraged by the legislature, or puffed into notice under the sanction of a quondam

But in my opinion, thofe are properly termed quack medicines, which are either advertifed under fictitious names, or anonymoufly; thofe of which the compofition is unknown, and the compounders and venders of which, are either ignorant pretenders to medical knowledge, or men in fituations or profeffions in life totally remote from the profeffion of phyfic.

Under the burden of medicines thus obtruded on the world, do the public at this moment groan; and not only the newfpapers and other periodical publications are filled with advertifements, recommending thefe doubtful and dreadful compounds, but every corner of the ftreets is furnifhed with diftributors, who load the paffengers with pamphlets and handbills, promifing, in the ftrongeft terms of affertion, infallible relief to the difeafed in all cafes; and inviting them to fwallow, for the cure of diforders fo very different in their natures as to make it obvious, on a moment's reflection, to all but the groffly ignorant, to whom, indeed, thefe delufive applications are particularly directed. Such is the credulity of mankind, that they fuffer themfelves to be duped by fuch barefaced artifices, and depending on a catalogue of pretended cures (into the truth of which they never give themfelves the trouble to enquire), they add to the number of the deceived, and, without regard to habit, conftitution, age, or fex, dofe themfelves with pills, potions, and powders, merely on the credit of their fabricated atteftations; and, at the expence of their health, and danger of their lives, minifter to the fraud and avarice of thofe retailers of poifon; the profits of fome of whom are fo extravagant, as to fupport them in enormous magnificent town-houfes and country villas, fplendid equipages, trains of fervants, and all the appendages of rank and fortune: though fome of thefe felf-created doctors are felected from the loweft of the people, and we are well affured, that one in particular now flourifhes in this metropolis, and fits at eafe in his own carriage, who a very few years ago was employed in the ftable, and driving one of thofe coaches which ply in the ftreets for the convenience of the public.

But thefe pefts to fociety are not content with publifhing their miferable infertions in the newfpapers, &c. but volumes are daily obtruded on the public which many of the nominal authors are unable to read; by thefe means they perfuade the deluded multitude that the moft dangerous difeafes

*quondam* eminent phyfician, are any thing short of a "burlesque on the common sense of mankind," yet, as the anonymous author of this paper has suggested some useful hints for the suppression of quack medicines; and as this is a subject so intimately connected with the advancement of the science, and the safety of the individual, we could not, consistently with our professions, refuse this ingenious letter a place in our Journal. W.

diseases are removed, and the most desperate cases cured, by these balsams of life and health, each of which, according to the pompous accounts of those hireling eulogists, possesses all the power of the whole of the materia medica, and is alone sufficient to prolong life to a period of years beyond that which has been allotted to the race of man; and we are sorry to add, that shops of reputation are to be found, base enough to participate in these practices of infamy, and to share in the price of their fellow-creatures' destruction.—That evils of such magnitude should be suffered to pass unnoticed, and that the lives of such numbers of useful members of society should be thus suffered to be trifled with and sported away, without the interposition of the legislature, in most other instances equally careful of property and life, and still permit poison, in a thousand forms, to be daily administered without check or controul, and that no benevolent member of the great councils of the nation should turn his attention to an object of such importance, is sufficient to excite our wonder and astonishment; and still more surprizing is it, that the learned body, to whom the practice of physic is legally committed, and whose abilities are as extensive as the powers with which they are invested, should suffer such enormities to proceed. By their intervention, either by an application to parliament to stop the progress of such alarming practices, or by exposing the compositions and effects of the several quack medicines offered to the public, detecting the falsity of the pretended cures performed by them, and exhibiting catalogues of the injuries sustained by those numerous individuals, whose lives have been destroyed or rendered comfortless by this unwarrantable traffic.—Surely the universal silence of the faculty on these concerns, seems to countenance an insinuation which we are well persuaded is unjust, "That the regular professors of physic are interested in the dissemination of these spurious nostrums, the suppression of which would lessen the progress of disease, and of course, diminish the number of patients who are ultimately compelled to seek relief from them, for the disorders brought on by quacks, mountebanks, empirics, &c."

If we might venture to offer a few hints on a subject apparently of so much consequence, we would submit to the legislature the propriety of erecting a public board, composed of the most eminent physicians, for the examination, analyzation, and approbation, of every new medicine, before an advertisement should be admitted into any newspaper or other periodical publication, and before it should be vended in any manner whatever.

To this board those who had made any medical improvement should apply, and state, on oath, the ingredients and composition of the proposed medicine, and the disorders to which it is meant to be applied; and if the members of the board ſhould think it may probably produce the propoſed effects, they ſhould immediately underwrite ſuch opinion on the advertiſements intended to be publiſhed, and under that ſanction the medicine ſhould be publicly ſold.

That the ſame board ſhould carefully examine into the truth of all caſes and atteſtations of cures publiſhed in ſupport of any advertiſed medicine; and in caſe any of them ſhould be diſcovered to be fraudulently fabricated, to declare the ſame by public advertiſement, in order to prevent the continuance of the impoſition; and we may venture to aſſert, that the members of the College of Phyſicians would be happy to execute an office ſo eſſential to the public benefit, without the ſmalleſt view of private emolument.

But we are well aware, that the plan we have propoſed is extremely imperfect, and the execution of it in its preſent form would be attended with many difficultie. We can only plead in excuſe for offering it, our zeal to remedy an increaſing evil, and our hope, that our humble attempt may induce ſome much abler writer to ſuggeſt a plan, better calculated to anſwer the important end for which it is deſigned.

