# Errata

**Published:** 1799-06

**Authors:** 


No. IV. p. 339, l. 12, for *life, and still permit poison in a thousand forms*, read *life;—that poison in a thousand forms should still be permitted.*

——— ibid. l. 24, after the word *traffic*, add, *the evil might be remedied.*

